# Attention to speech modulates distortion product otoacoustic emissions evoked by speech-derived stimuli in humans

**DOI:** 10.3389/fnins.2026.1756386

**Published:** 2026-03-02

**Authors:** Janna Steinebach, Tobias Reichenbach

**Affiliations:** Department Artificial Intelligence in Biomedical Engineering, Friedrich-Alexander-Universität Erlangen-Nürnberg, Erlangen, Germany

**Keywords:** auditory attention, distortion products, efferent feedback, inner ear biology, MOC system, otoacoustic emissions, speech processing

## Abstract

Humans are remarkably skilled at understanding speech in noisy environments. While segregation of different audio streams is mostly accomplished in the auditory cortex, neural feedback connections run from the cortex to the brainstem and to the cochlea. The latter organ not only houses the mechanosensitive hair cells, but also possesses an active process enabling it to amplify sound in a frequency-dependent manner. A physiological correlate of the active process are distortion-product otoacoustic emissions (DPOAEs) that can be measured non-invasively from the ear canal. Here we employed speech-like DPOAEs, measured in response to stimuli derived from natural human speech and thus reflecting the harmonic spectral structure of voiced speech. We show that these emissions are modulated by selective attention to one of two competing voices, as well as by intermodal attention. Specifically, speech-like DPOAEs evoked by stimuli related to resolved harmonics of a voice were significantly reduced when that voice was attended compared to when it was ignored. No such effect was observed for stimuli related to unresolved harmonics of the target voice when the competing voice's harmonics in that range were unresolved as well, indicating that attentional modulation is specific to those components of voiced speech that are spectrally resolved. Our findings support the hypothesis that the cochlea's active process already shapes selective attention to speech in noise. Moreover, the speech-like DPOAEs that we developed open up further possibilities for investigating the contribution of the cochlear active process to auditory scene analysis in naturalistic settings.

## 1 Introduction

Understanding speech in noisy environments is an important yet highly complex human ability. In crowded settings such as restaurants or family gatherings, selective auditory attention enables listeners to focus on a single speaker while filtering out competing voices—also known as the cocktail party effect (McDermott, 2009; Cherry, 1953). This ability is essential for many aspects of social participation, but is vulnerable to hearing damage: many people with hearing impairment complain of difficulty understanding speech in background noise, even when using hearing aids (Plomp, 1978).

The neural machinery behind selective auditory attention, including attention to speech, has been extensively studied at the level of the cerebral cortex (Pugh et al., 1996; Lakatos et al., 2013; Mesgarani and Chang, 2012; Ding and Simon, 2012; Horton et al., 2013). However, anatomical and physiological evidence points to substantial descending feedback from the auditory cortex to the auditory brainstem and to the cochlea (Huffman and Henson, 1990; Pickles, 1988; Winer et al., 1998). Through these neural feedback loops, subcortical processing centers may contribute to accomplishing the cocktail party effect. Several studies have indeed found that neural responses from the brainstem, in particular frequency-following responses, can be modulated by selective auditory attention, although others did not find an attentional effect (Galbraith et al., 2003; Forte et al., 2017; Etard et al., 2019; Strauss et al., 2025; Stoll et al., 2025; Xie, 2025).

The cochlea—the sensory organ of hearing in which sound vibrations are converted into electrical signals—may already contribute to selective auditory attention as well. This fascinating organ spatially decomposes a complex sound such as speech into its individual frequency components, following a tonotopic map in which high frequencies are detected near the organ's base, and lower frequencies progressively further toward the apex (Robles and Ruggero, 2001; Reichenbach and Hudspeth, 2014).

In addition, the cochlea possesses an active process through which it amplifies weak sounds (Dallos, 1992; Hudspeth, 2014). This mechanical amplification is provided by outer hair cells and can be reduced through activation of the medial olivocochlear (MOC) fibers—efferent connections that innervate the outer hair cells (Guinan, 2006; Lopez-Poveda, 2018). Because each MOC fiber is tuned to a narrow frequency band, and because the innervation of the cochlea by these fibers displays a tonotopic arrangement, the gain of the active process can potentially be regulated by the brain in a frequency-dependent manner.

The active process is accompanied by a nonlinear response that gives rise to otoacoustic emissions (OAEs). These can be recorded from the ear canal and serve as a non-invasive measure of the amplification gain. OAEs have indeed been used to assess the contribution of the cochlea to selective attention, although with inconclusive results (Meric and Collet, 1992; Michie et al., 1996; Walsh et al., 2015; Smith et al., 2012; Beim et al., 2018; Francis et al., 2018; Wittekindt et al., 2014). A limitation of these studies was that they either did not involve naturalistic sounds such as speech that facilitate attention, or that they elicited OAEs in a manner that was not directly related to the auditory signal that the participants were asked to attend.

Computational models have shown that selective attention to a speech signal in noise may be supported by frequency-specific modulation of the cochlear active process (Messing et al., 2009; Clark et al., 2012). Most parts of speech are voiced, with the energy carried by the fundamental frequency and its many higher harmonics. The lower harmonics, up to the 10th, can be spatially resolved in the cochlea, that is, they cause peaks at significantly distinct locations (Bernstein and Oxenham, 2003; Pit, 2005; Micheyl and Oxenham, 2007; Carcagno and Plack, 2011).

A compelling hypothesis is that the cochlear amplifier selectively enhances the resolved harmonics of a target speech and suppresses the spectral bands that lie inbetween. This mechanisms would thus reduce background noise already at the level of cochlear activity. Because unresolved harmonics cannot be spatially differentiated in the cochlea, this mechanism should not be able to operate for these.

Here, we set out to test this hypothesis. We employed distortion product otoacoustic emissions that were evoked by certain higher harmonics of the voiced parts of speech (speech-like DPOAEs). As the fundamental frequency of natural speech varies over time, the stimuli used to generate speech-like DPOAEs, as well as the DPOAEs themselves, were not pure tones, but instead had instantaneous frequencies that varied over time in proportion to the fundamental frequency of the source signal. The amplitude of the stimuli varied as well, in particular, it was zero during voiceless parts of the speech signal or during silences. We recently developed and corroborated this approach (Saiz-Alía et al., 2021).

We elicited and recorded speech-like DPOAEs from one ear while two competing talkers were presented to the contralateral ear. Subjects were instructed to attend either one of the two talkers or to read a text in front of them (visual attention). We then evaluated how the speech-like DPOAEs, in particular those related to resolved and unresolved harmonics, were affected by the attentional focus.

## 2 Materials and methods

### 2.1 Experimental design

We utilized a single- and a competing-speaker paradigm. In the single-speaker recordings, an audiobook either spoken by a female or by a male voice was presented to the right ear of the participant. Subjects were asked to focus their attention on the single voice.

**Figure 1:**
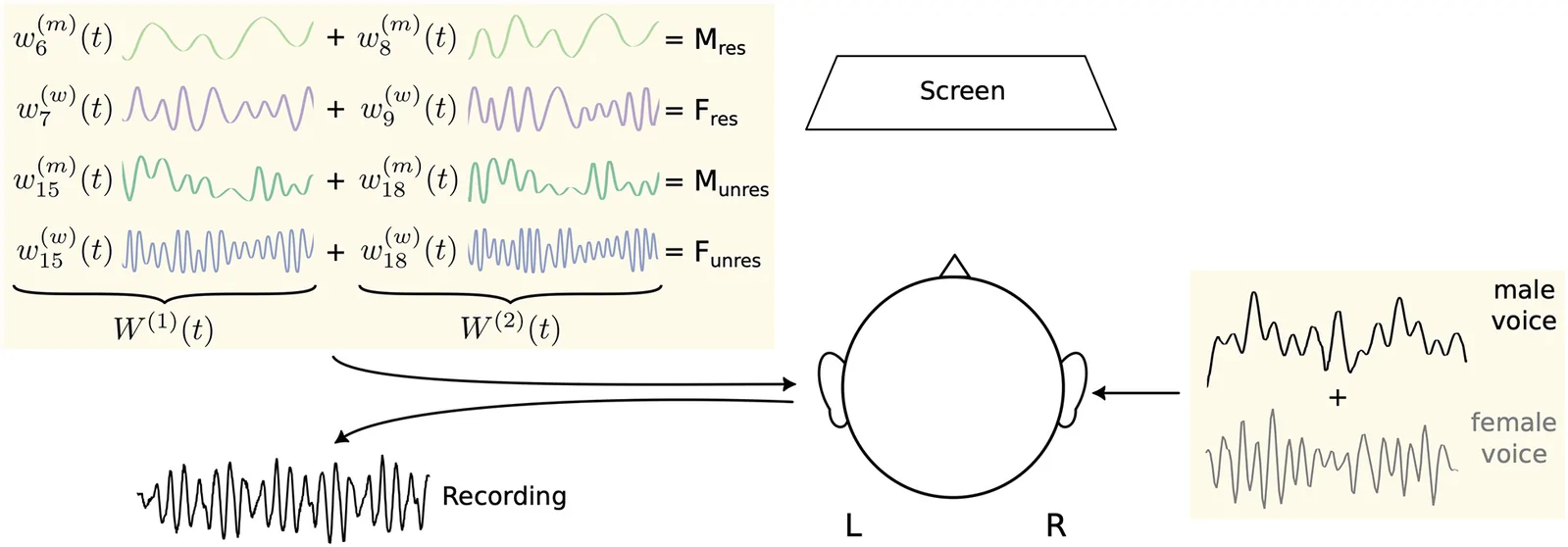
Experimental setup. Two audiobooks, one spoken by a women with fundamental frequency $f_{0}^{\left(w\right)}\left(t\right)$ and one narrated by a man with fundamental frequency $f_{0}^{\left(m\right)}\left(t\right)$, were simultaneously presented to the right ear. Speech-like DPOAEs were recorded from the left ear. They were elicited by four pairs of waveforms. The first stimulus pair, *M*_res_, consisted of two waveforms $w_{6}^{\left(m\right)}\left(t\right)$, $w_{8}^{\left(m\right)}\left(t\right)$ based on resolved harmonics of the male voice [frequencies $6f_{0}^{\left(m\right)}\left(t\right)$ and $8f_{0}^{\left(m\right)}\left(t\right)$]. The second stimulus pair, *F*_res_, comprised two waveforms $w_{7}^{\left(w\right)}\left(t\right)$, $w_{9}^{\left(w\right)}\left(t\right)$ following resolved harmonics of the female voice [frequencies $7f_{0}^{\left(w\right)}\left(t\right)$ and $9f_{0}^{\left(w\right)}\left(t\right)$]. The third and fourth stimulus pair, *M*_unres_ and *F*_unres_, were waveforms $w_{15}^{\left(m\right)}\left(t\right)$, $w_{18}^{\left(m\right)}\left(t\right)$ resp. $w_{15}^{\left(w\right)}\left(t\right)$, $w_{18}^{\left(w\right)}\left(t\right)$ derived from unresolved harmonics of the male resp. female voice [frequencies $15f_{0}^{\left(m\right)}\left(t\right)$ and $18f_{0}^{\left(m\right)}\left(t\right)$ resp. $15f_{0}^{\left(w\right)}\left(t\right)$ and $18f_{0}^{\left(w\right)}\left(t\right)$]. Participants directed their attention either to one of the two talkers or visually to a continuously presented story on a screen.

In the competing-speaker scenario, two audiobooks, one spoken by a women and the other by a man, were added together and presented to each subject's right ear (Figure 1). Subjects were then instructed to attend either the female or the male voice. In the following, we refer to these two attentional conditions as attended female voice (Att. F) and attended male voice (Att. M). To test effects of intermodal attention, a visual distractor was introduced as well and attended upon prompt; participants then read a story displayed in segments on a screen while ignoring both audio streams. This attentional condition will be referred to as attended visual distractor (Att. V).

Speech-like DPOAEs were elicited by waveforms derived from the speech signals. To enable clean simultaneous measurement of four different speech-like DPOAEs, these were evoked and measured from the contralateral, i.e. the left, ear.

The presentation of the audiobooks together with the recordings of the speech-like DPOAEs were segmented into two-minute trials, each followed by three comprehension questions and a rating of perceived mental effort to ensure task engagement. For the auditory conditions (attending the female resp. male voice), mental effort was equivalent to the listening effort. In order to include assessment of the effort for the third, i.e. the visual, condition, the term mental effort was chosen.

### 2.2 Participants

Speech-like DPOAE measurements were conducted with *N* = 40 participants (21 female, 19 male), aged 18–31 years (mean age ± SD: 25 ± 3 years). Inclusion criteria were right-handedness, native German proficiency, and the absence of neurological or hearing impairments. One subject was excluded since their comprehension score was below chance level, and another due to a faulty microphone recording. 38 participants were thus included in the final analysis.

All procedures were approved by the ethics board of the University Hospital Erlangen (registration 133-12B) and were conducted in accordance with institutional regulations. Informed consent was obtained from all participants.

### 2.3 Speech signals

Two audiobooks were synthesized with a text-to-speech engine (ElevenLab, U.S.A.) using the voice “Matilda” for the female speaker and the voice “Brian” for the male one (ElevenLabs, n.d.). We chose voice parameters that yielded a large separation between the fundamental frequencies of the two voices, which resulted in an average fundamental frequency of ${\bar{f}}_{0}^{\left(w\right)}=195\pm 40$ Hz for the female voice and ${\bar{f}}_{0}^{\left(m\right)}=90\pm 20$ Hz for the male voice (mean ± SD).

The texts for the audiobooks were taken from “Eine Frau erlebt die Polarnacht” (Ritter, 2016) (Book A) and from “Darum” (Glattauer, 2018) (Book B). Each text was synthesized with both the female and the male voice.

The amplitudes of the digital waveforms representing the speech signals were normalized and scaled such that the root-mean-square amplitude was (2.0 ± 0.1) × 10^−3^ for both the male- and female-spoken texts. Using PsychoPy (Peirce et al., 2019), the presentation level of the speech stimuli was adjusted such that it reached an average sound pressure level of 57 dB SPL over the two-minute segments.

### 2.4 Visual distractor

The visual distractor consisted of text excerpts from a third book, “Frau Ella” (Beckerhoff, 2022) (Book C). The text was rendered as a video in which short paragraphs appeared word by word at a comfortable reading pace and were displayed centrally on a computer screen.

### 2.5 Stimuli for eliciting speech-like DPOAEs

Pure-tone DPOAEs were elicited by two primary frequencies, *f*_1_ and *f*_2_. The lower-sideband cubic distortion product 2*f*_1_−*f*_2_ is the strongest, and is maximal at a ratio of *f*_2_/*f*_1_≈1.2. For its measurement we employed *f*_1_ = 1 kHz and *f*_2_ = 1.2 kHz.

The stimuli to elicit speech-like DPOAEs were computed using an approach that we developed recently (Saiz-Alía et al., 2021).

For the voiced segments of each speech signal, we first computed the fundamental waveform *w*_0_(*t*), which follows the time-varying fundamental frequency *f*_0_(*t*) of the source signal. This was achieved by applying a zero-phase, sixth-order IIR bandpass filter centered on the mean fundamental frequency $\bar{f_{0}}$, with corner frequencies of ±0.5 standard deviations around $\bar{f_{0}}$. The mean fundamental frequency was estimated using the probabilistic YIN algorithm implemented in the librosa library (McFee et al., 2025). The fundamental waveform *w*_0_(*t*) was normalized by z-scoring.

Based on *w*_0_(*t*), waveforms for the harmonic overtones *n* and *m* (*n*<*m*) were constructed such that their instantaneous frequencies equaled *nf*_0_(*t*) and *mf*_0_(*t*), respectively. To do so, we computed the analytic representation of the fundamental waveform using the Hilbert transform *H*[*w*_0_(*t*)]:


$$\begin{array}{l} W_{0}\left(t\right)=w_{0}\left(t\right)+i\cdot H\left[w_{0}\left(t\right)\right]. \end{array}$$ (1)

The fundamental waveform can then be expressed as the real part of the complex signal:


$$\begin{array}{l} w_{0}\left(t\right)=ℜ\left[A\left(t\right)\cdot e^{i\Phi \left(t\right)}\right], \end{array}$$ (2)

in which $A\left(t\right)=|W_{0}\left(t\right)|$ denotes the signal amplitude, and $\Phi \left(t\right)\;=\;\arg\left[W_{0}\left(t\right)\right]$ specifies its instantaneous phase.

Harmonic waveforms *w*_*n*_(*t*) and *w*_*m*_(*t*) were obtained by multiplying the phase Φ(*t*) by the desired harmonic number and taking the real part:


$$\begin{array}{l} w_{n}\left(t\right)=ℜ\left[A\left(t\right)\cdot e^{i\Phi \left(t\right)\cdot n}\right]\text{and }w_{m}\left(t\right)=ℜ\left[A\left(t\right)\cdot e^{i\Phi \left(t\right)\cdot m}\right]. \end{array}$$ (3)

The instantaneous frequencies of the two elicitor waveforms are thus *nf*_0_(*t*) and *mf*_0_(*t*). Consequently, the lower-sideband cubic distortion product they generate exhibits an instantaneous frequency of (2*n*−*m*)*f*_0_(*t*), corresponding to the waveform *w*_2*n*−*m*_(*t*). The resulting speech-like DPOAE was identified by cross-correlating *w*_2*n*−*m*_(*t*) with the microphone recording.

To assess attentional modulation of cochlear activity at both resolved and unresolved harmonics, we designed four pairs of stimulus waveforms: (1) the stimulus *F*_res_: two waveforms $w_{7}^{\left(w\right)}\left(t\right)$, $w_{9}^{\left(w\right)}\left(t\right)$ derived from resolved harmonics of the female voice, (2) the stimulus *F*_unres_: two waveforms $w_{15}^{\left(w\right)}\left(t\right)$, $w_{18}^{\left(w\right)}\left(t\right)$ derived from unresolved harmonics of the female voice, (3) the stimulus *M*_res_: two waveforms $w_{6}^{\left(m\right)}\left(t\right)$, $w_{8}^{\left(m\right)}\left(t\right)$ derived from resolved harmonics of the male voice, and (4) the stimulus *M*_unres_: two waveforms $w_{15}^{\left(m\right)}\left(t\right)$, $w_{18}^{\left(m\right)}\left(t\right)$ derived from unresolved harmonics of the male voice (Table 1).

**Table 1:** The harmonic numbers *n* and *m* for the different stimuli, with the harmonic index 2*n*−*m* of the resulting lower-sideband cubic distortion product and the ratio *m*/*n*. ${\bar{f}}_{w_{n}}$, ${\bar{f}}_{w_{m}}$ and ${\bar{f}}_{w_{2n-m}}$ denote the average frequencies of the waveforms *w*_*n*_(*t*), *w*_*m*_(*t*) and *w*_2*n*−*m*_(*t*) as (mean ± SD) across all 2 min segments.

**Stimulus type**	***n***	***m***	**2*n*−*m***	***m*/*n***	**${\bar{f}}_{w_{n}}$ (Hz)**	**${\bar{f}}_{w_{m}}$ (Hz)**	**${\bar{f}}_{w_{2n-m}}$ (Hz)**
*F*_res_	7	9	5	1.29	1,360 ± 73	1,750 ± 94	970 ± 50
*F*_unres_	15	18	12	1.2	2,910 ± 158	3,500 ± 190	2,320 ± 120
*M*_res_	6	8	4	1.33	530 ± 37	710 ± 49	350 ± 22
*M*_unres_	15	18	12	1.2	1,330 ± 91	1,600 ± 109	1,050 ± 67

Importantly, the terms “resolved” and “unresolved” refer here to the harmonic structure of the speech signal presented to the right ear. In contrast, in the left, contralateral, ear used for speech-like DPOAE recording, the two waveforms constituting each stimulus pair [e.g., $w_{15}^{\left(m\right)}\left(t\right)$ and $w_{18}^{\left(m\right)}\left(t\right)$ representing the 15th resp. 18th harmonic overtone of the speech signal] are separated by a similar frequency ratio as the lower-order pairs and are therefore comparably resolved by cochlear filtering. Thus, the distinction between resolved and unresolved stimuli does not pertain to the separability of the harmonic numbers of the stimulus waveforms themselves, but to whether the surrounding harmonic components in the speech signal are resolved or not. For the unresolved harmonics, multiple adjacent harmonics fall within a single cochlear filter, resulting in broad excitation of the cochlear region corresponding to the DPOAE generation site and, consequently, a different pattern of MOC-mediated modulation.

We employed different harmonics *n* and *m* for the resolved harmonics of the female and the male voice in order to avoid unwanted correlations between the stimulus waveforms and the DPOAE waveforms. Moreover, we tried to achieve a ratio of *m*/*n*≈1.2 for optimal distortion product generation. For unresolved harmonics, the frequency spacing was sufficiently large to allow the same harmonic pairs to be used for male- and female-related stimuli without inducing unwanted correlations between the speech-like DPOAE and stimulus waveforms.

As a consequence of these design choices, the frequency ranges of the resolved harmonics of the female voice partially overlap with those of the unresolved harmonics of the male voice (Table 1). This overlap arises from the different fundamental frequencies of the male and female voices and the need to balance harmonic resolvability with the constraints mentioned above. The implications of this spectral overlap for the interpretation of the results are addressed in the Discussion.

For the simultaneous presentation of all four harmonic pairs, all waveforms corresponding to the lower harmonic number *n* were summed to form the waveform *W*^(1)^(*t*), and all waveforms corresponding to the higher harmonic number *m* were summed separately to form the waveform *W*^(2)^(*t*). These two waveforms were delivered to the ear canal via two independent loudspeakers.

### 2.6 Experimental setup

Experiments were conducted in a sound-proof, semi-anechoic chamber. Stimulus presentation and data acquisition were automated through PsychoPy (Peirce et al., 2019). Instructions were displayed on a screen; responses were given via mouse click.

The sound stimuli were presented at 44.1 kHz using a high-performance sound card (RME Fireface 802) and delivered through an extended-bandwidth otoacoustic measurement system (ER10X, Etymotics, U.S.A.), equipped with one microphone and three speakers per ear. Custom ear tips ensured optimal probe fit. Audiobooks were presented to the right ear while stimuli presentation and speech-like DPOAE recordings were conducted in the left ear. For each stimulus, the two waveforms *W*^(1)^(*t*) and *W*^(2)^(*t*), each consisting of the sum of the four harmonic waveforms corresponding to the lower and higher harmonic numbers *n* and *m* respectively, were played through different speakers to avoid hardware-induced distortion.

Stimuli were delivered directly into the ear canal. The presentation level was adjusted so that, when averaged across each trial of approximately two minutes, the resulting mean sound pressure level in the ear canal was 57 dB SPL. All participants reported this level as comfortable. It was kept below the levels at which the middle-ear muscle reflex is typically elicited (Jennings, 2021; Trevino et al., 2023).

### 2.7 Experimental routine

Each story segment lasted about two minutes. After each such two-minute trial, participants answered three comprehension questions and rated the perceived mental effort on a 13-point Likert scale.

The experiment began with a two-minute pure-tone DPOAE measurement to verify DPOAE detectability. DPOAEs could be recorded in all participants.

Next, speech-like DPOAEs were recorded in a single-speaker scenario, using either the male and the female voice in isolation. Speech-like DPOAEs to both the resolved and the unresolved harmonics of the corresponding voice were measured simultaneously to confirm that both speech-like DPOAEs could be measured concurrently.

The main part of the experiment consisted of a competing-speaker scenario in which both the female and the male voice were presented simultaneously. Participants were instructed to direct their attention either to the female speaker (Att. F), to the male speaker (Att. M), or to the visual task (Att. V). The target audio thereby always contained the story of Book A, spoken either by the male or the female voice, allowing participants to follow a continuing story when switching attention between speakers. In the Att. V. condition, participants ignored both audio streams and focused on reading Book C, presented word-by-word on a monitor. During the two auditory-attention conditions (Att. F and Att. M), the text from Book C was also shown on the monitor; however, participants were allowed to choose whether to look at the text directly or at another fixed point on the screen, depending on whether they found looking at the text too distracting from the auditory task. During each of the conditions, the waveforms *W*^(1)^(*t*) and *W*^(2)^(*t*) comprising the four stimulus pairs *F*_res_, *F*_unres_, *M*_res_, and *M*_unres_ were presented to elicit the four respective speech-like DPOAEs.

To verify that the measurement equipment did not contribute to the speech-like DPOAEs, out-of-ear control measurements were conducted. The probe was placed outside the ear canal in the center of the recording room, with all reflective surfaces avoided to minimize acoustic feedback. No speech-like DPOAEs emerged in that case.

### 2.8 Analysis of speech-like DPOAEs

Hardware-induced delays were estimated per trial by cross-correlating the stimulus waveforms with the microphone recording. The recordings were corrected for the delays before further analysis.

Speech-like DPOAEs were computed by cross-correlating each of the four speech-like DPOAE waveforms *w*_2*n*−*m*_(*t*) with the microphone recording. To compensate for potential phase shifts between the otoacoustic emission and the waveform *w*_2*n*−*m*_(*t*), we computed the complex cross-correlation. Its real part corresponds to the correlation between the real component of the analytic representation of *w*_2*n*−*m*_(*t*) and the microphone signal, whereas the imaginary part corresponds to the correlation with the imaginary component of the analytic representation. The envelope of the complex cross-correlation was then obtained as the absolute value of the resulting complex-valued correlation.

Grand averages were computed by averaging the envelopes of the cross-correlations across all two-minute trials, distinguishing between the three attentional conditions and the four stimulus types. A peak in the grand average was considered significant if it exceeded the maximum of the noise. The noise level was thereby determined from the values of the envelope of the correlation coefficients at time lags of −750 to −70 ms and from 70 to 750 ms, that is, at delays at which no speech-like DPOAEs should occur.

To detect speech-like DPOAEs at the level of individual two-minute trials, the expected window for peak delays was defined as 1 ± 3 ms (mean ± SD), based on the grand averages of the stimuli *F*_res_ and *F*_unres_. Stimuli corresponding to the male voice were excluded for the determination of this window: the grand average for the *M*_res_ stimulus did not yield reliable results, and we wanted to prevent an imbalance between resolved and unresolved harmonics, such that we disregarded the stimulus *M*_res_ as well. A peak from an individual trial within this window was considered significant if it exceeded the 97th percentile of the noise. The percentage of significant trials was then computed for each stimulus type.

To compare speech-like DPOAEs across attentional conditions, the cross-correlation coefficients at the grand average peak delay were extracted for each two-minute trial, matched per stimulus type and attentional condition, and averaged per participant. Single-speaker comparisons used unpaired *t*- or Mann–Whitney *U* tests. For the data obtained from the competing-speaker scenario, paired *t*-tests or Wilcoxon signed-rank tests were used, with outlier exclusion when justified.

To evaluate differences between speech-like DPOAEs evoked by resolved and unresolved harmonics, peak delays per individual two-minute trials were extracted for all significant peaks, matched per stimulus type and condition and averaged per participant. Statistical comparisons used unpaired *t*- or Mann–Whitney *U* tests.

All *p*-values were corrected for multiple comparisons using the False Discovery Rate correction.

## 3 Results

### 3.1 Comprehension scores and mental effort

Speech comprehension was quantified as the percentage of correct answers, and mental effort was rated using mean values on a Likert scale from 1 to 13 (low to high). Values are given as (mean ± SD). Statistical significance was assessed using Wilcoxon signed-rank tests.

In the single-speaker measurements, comprehension scores were high: 97 ± 13% for the female voice and 96 ± 14% for the male voice, with no significant difference between them.

The comprehension scores were slightly lower in the competing-speaker condition: 95 ± 6% when attending the female voice, 91 ± 8% for the male voice, and 94 ± 7% when reading the text. Again, no significant differences were found, suggesting that all conditions were similarly comprehensible.

Regarding mental effort, the female and male voices were perceived as similarly demanding in the single-speaker condition, with ratings of 4.0 ± 2.3 vs. 4.4 ± 2.4 and no significant difference.

In the competing-speaker condition, perceived effort varied significantly depending on the attentional focus. Attending the visual distractor was rated easiest at 5.7 ± 2.2; lower than when attending the male voice (*p* < 0.001) and lower than when attending the female voice (*p* < 0.05). In contrast, attending the male voice was rated hardest at 7.3 ± 1.7; with *p* < 0.001 when compared to other conditions. Attending the female voice fell in between at a value of 6.5 ± 1.8.

### 3.2 Measurement of speech-like DPOAEs

To measure a particular speech-like DPOAE, we computed a waveform *w*_2*n*−*m*_(*t*) that corresponded to the lower-sideband cubic distortion product of the pair of harmonics that was used for stimulation. As an example, for the stimulus *F*_res_ we utilized waveforms $w_{7}^{\left(w\right)}\left(t\right)$ and $w_{9}^{\left(w\right)}\left(t\right)$, yielding $w_{5}^{\left(w\right)}\left(t\right)$ as the lower sideband cubic distortion product.

**Figure 2:**
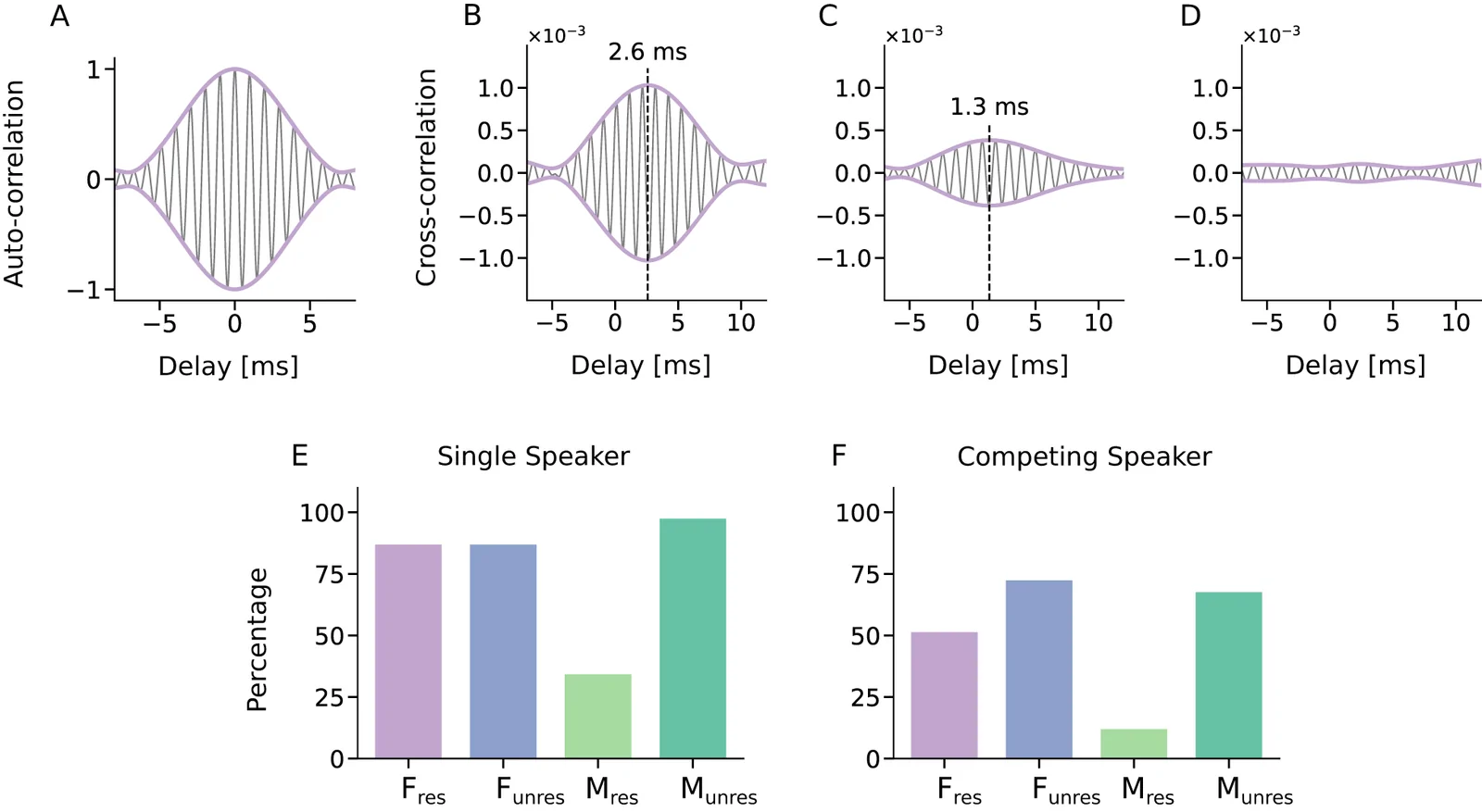
Recording of speech-like DPOAEs. Speech-like DPOAEs were measured through cross-correlating the expected DPOAE waveform *w*_2*n*−*m*_(*t*) with the microphone signal. **(A)** The auto-correlation of the speech-like DPAOE waveform *w*_5_(*t*) of the *F*_res_ stimulus gives an estimate for the morphology of the expected peak. The envelope (purple) of the cross-correlation (black) peaks at 0 ms, as required for an autocorrelation. **(B, C)** The correlation of the waveform *w*_5_(*t*) of the *F*_res_ stimulus with the microphone recording, for two individual trials, exhibited a peak at a short delay (dashed line) that showed the presence of the speech-like DPOAE at this delay. **(D)** In contrast, if no peak emerged, the speech-like DPOAE could not be measured for that particular trial. The exemplary recordings show results from a single two-minute trial in the single-speaker scenario, for the *F*_res_ stimulus and for different subjects. **(E, F)** Speech-like DPOAEs could be detected in most trials both in the single-speaker and in the competing-speaker scenario, except for the stimulus *M*_res_.

The speech-like DPOAE waveform *w*_2*n*−*m*_ was then cross-correlated with the microphone recording (Figures 2A–D). To obtain a sense of the expected shape of the cross-correlation, we first computed the auto-correlation of a waveform *w*_2*n*−*m*_ (Figure 2A). As expected, this showed a single peak at zero latency, which was particularly apparent in the autocorrelation's envelope.

We then computed the envelope of the cross-correlation of the waveform *w*_2*n*−*m*_ with the microphone recording to obtain, in most subjects, a single peak at a delay between 0 − 3 ms. A peak can be interpreted as a successful measurement of a speech-like DPOAE, with a delay that corresponds to that of the peak. Examples of individual trials with large and moderate peak amplitudes, as well as a trial without a significant peak, are shown in Figures 2B–D, illustrating the variability in speech-like DPOAE morphology. These examples further show that the width of the cross-correlation parallels that of the autocorrelation, indicating that the temporal resolution is limited by the autocorrelation, not by additional temporal jitter in the emissions.

To assess how well the speech-like DPOAEs for the different stimuli could be measured, we computed the percentage of trials per stimulus type that yielded significant peaks in the cross-correlation. In the single-speaker scenario, the three stimuli *F*_res_, *F*_unres_, and *M*_unres_ all yielded significant speech-like DPOAEs in over 85% of single-speaker trials (Figure 2D). However, the stimulus *M*_res_ performed notably worse, with only 35% of trials showing a significant speech-like DPOAE.

A similar pattern emerged in the competing-speaker scenario (Figure 2E): the stimuli *F*_unres_ and *M*_unres_ performed well with speech-like DPOAEs detectable in about 70% of the trials, the stimulus *F*_res_ slightly lower, with about 50% of trials yielding significant speech-like DPOAEs, and *M*_res_ remained poor with only about 12% of trials producing a significant measurement.

To further compare the speech-like DPOAEs evoked by the different stimuli, we averaged the envelopes of the complex cross-correlations across trials and subjects, yielding grand averages.

**Figure 3:**
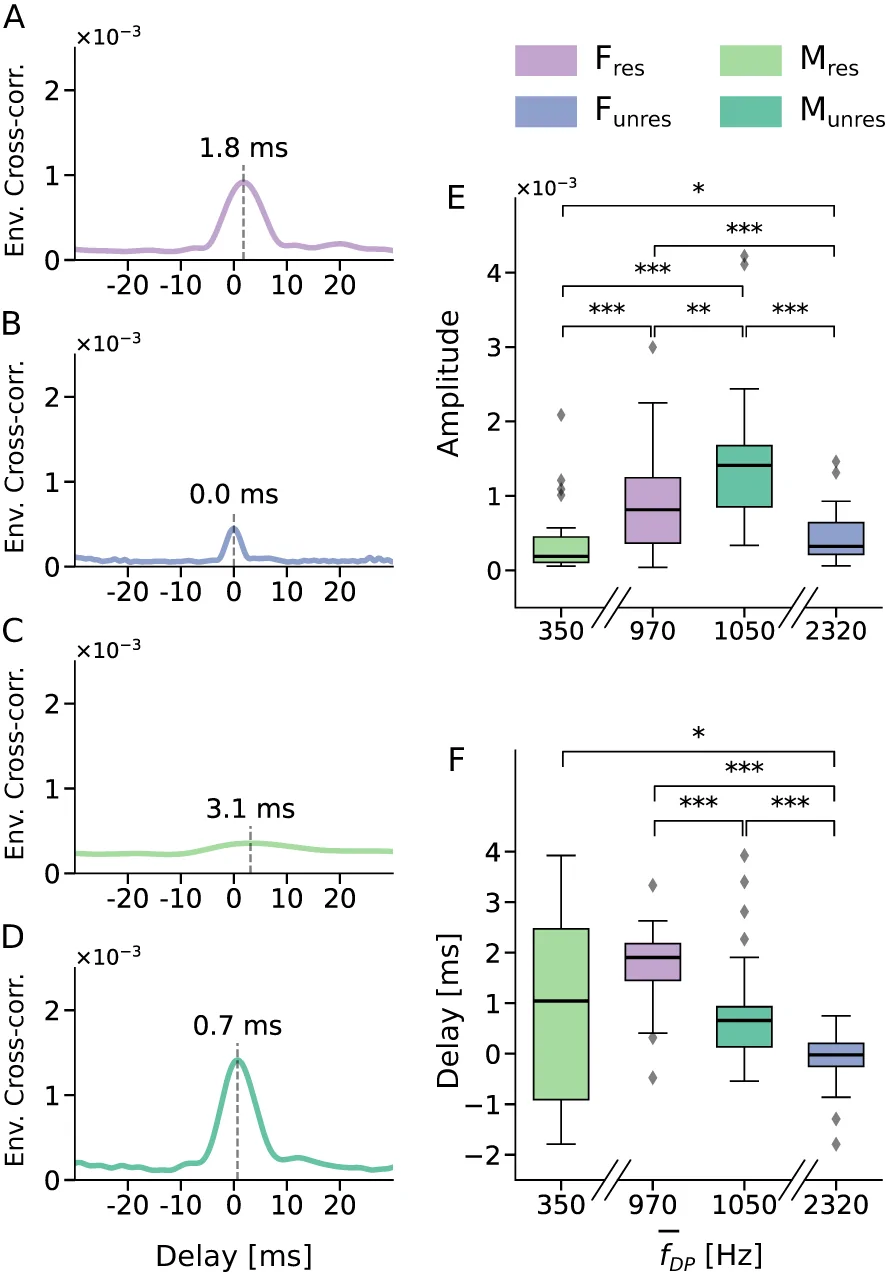
Speech-like DPOAEs in the single-speaker scenario. **(A–D)** Grand averages of the envelopes of the complex cross-correlations for the different stimuli. All peaks except the one for the *M*_res_ stimulus were clearly visible. The delays of the peaks are marked by dashed lines. **(E)** Speech-like DPOAE amplitudes varied significantly across stimulus types. They displayed an inverted U when plotted against the average frequency ${\bar{f}}_{DP}$ of the expected speech-like DPOAE waveforms *w*_2*n*−*m*_, with the largest amplitudes emerging for speech-like DPOAEs with average frequencies around 1 kHz. (F) Peak delays of individual trials differed significantly across stimulus types. The variation in delays for the *M*_*res*_ stimulus was high due to the low number of significant peaks. Statistical significance is indicated as ^*^ (*p* < 0.05), ^**^ (*p* < 0.01), ^***^ (*p* < 0.001).

In the single-speaker scenario, all stimulus types produced significant peaks, but with considerable variation in the amplitudes, that is, the values of the envelopes of the cross-correlations at the peak (Figure 3E). The speech-like DPOAE for the stimulus *M*_res_, with an average frequency of 350 Hz, had the smallest amplitude. The highest amplitudes emerged for the stimuli *F*_res_ and *M*_unres_, both of which produced speech-like DPOAEs with average frequencies around 1 kHz.

The delays of the speech-like DPOAEs also varied across the four stimulus types (Figure 3F). The shortest delay was observed for the *F*_unres_ stimulus at 0 ms, while the longest delay occurred for the *M*_res_ stimulus at 3.1 ms. For the *M*_res_ stimulus, peak delays showed substantial variability across participants. This variability was likely due to the low number of significant peaks for this stimulus type (Figures 2D, E), further underscoring the limited interpretability of the corresponding results.

Due to the small amplitude and the low rate of significant peaks observed, the stimulus *M*_res_ was excluded from further analysis.

**Figure 4:**
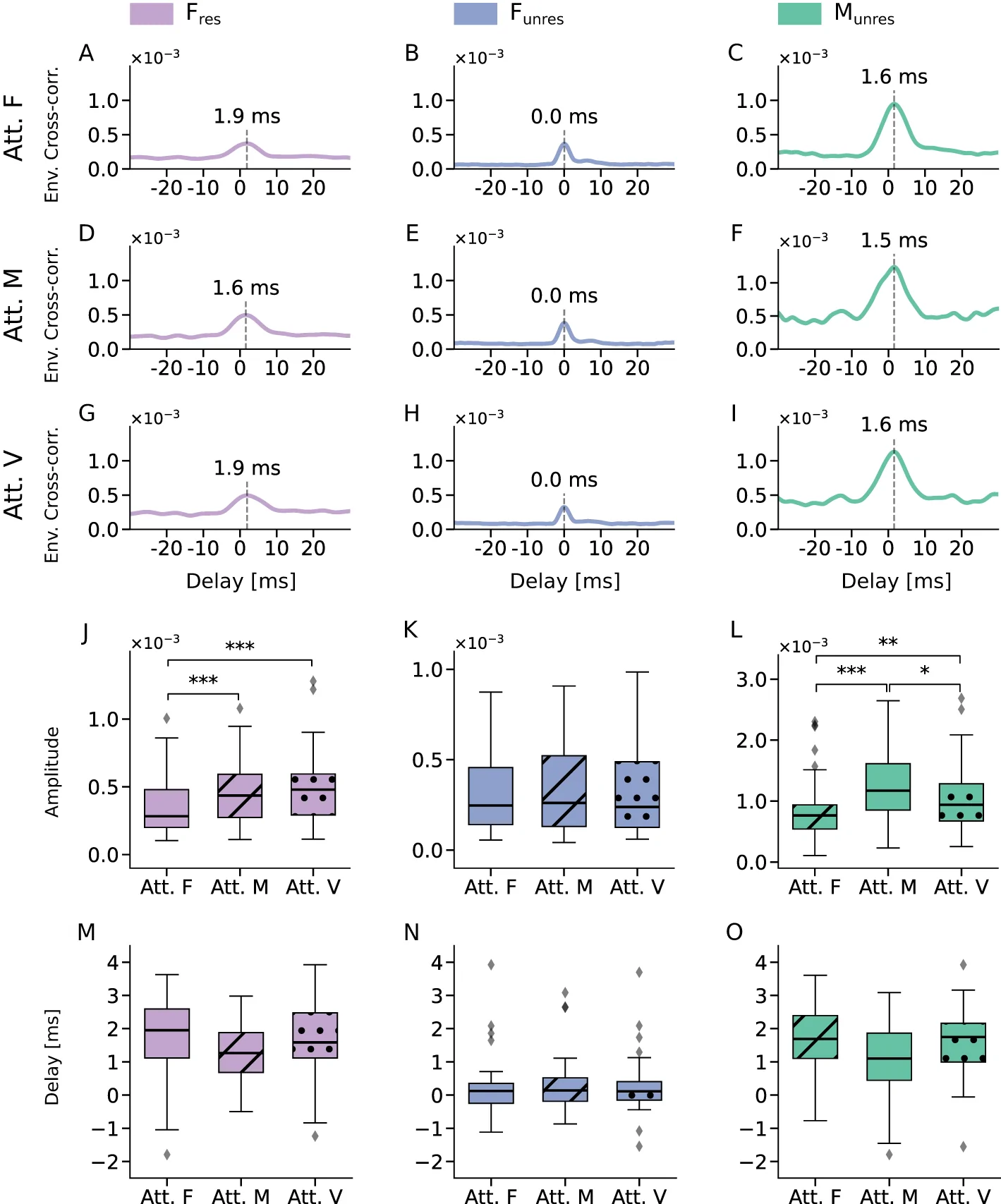
Attentional modulation of speech-like DPOAEs. **(A–I)** Envelopes of the complex cross-correlations of speech-like DPOAEs evoked by the three different stimuli *F*_res_, *F*_unres_, and *M*_unres_, in the three different attentional conditions attended female voice (Att. F), attended male voice (Att. M), and attended visual distractor (Att. V.). All showed clear peaks at short delays (dashed lines). **(J–L)** Comparison of the peak amplitudes yielded attentional effects for the *F*_res_ stimulus and the *M*_unres_ stimulus, but not for the stimulus *F*_unres_. **(M–O)** The peak delays were not affected by the attentional focus, for neither of the three stimulus types. Statistical significance is indicated as ^*^ (*p* < 0.05), ^**^ (*p* < 0.01), ^***^ (*p* < 0.001).

For competing-speaker trials, the remaining stimuli (*F*_res_, *F*_unres_, *M*_unres_) produced significant peaks in all three attentional conditions (Figure 4A–I). While the delays of the peaks remained stable across attentional conditions, they continued to vary between stimulus types.

Out-of-ear control measurements did not yield significant peaks in the grand average for any stimulus type in single- or competing-speaker trials, confirming the absence of measurable distortion products when the probe was placed outside the ear canal.

### 3.3 Attentional modulation of speech-like DPOAEs

To assess effects of attentional focus on the speech-like DPOAEs in the competing-speaker scenario, we characterized the latter through both the delay of the peak in the complex cross-correlation and the amplitude, that is, the value of the envelope of the complex cross-correlation at the grand average peak delay.

We first quantified the influence of attention on the amplitude of the emissions. We started with the stimulus *F*_res_, that is, by assessing the speech-like DPOAEs evoked by resolved harmonics of the female voice (Figure 4J). We found a significantly lower amplitude when the female speaker was attended (Att. F) than when the male speaker was attended (Att. M). The difference was highly statistically significant, with a *p*-value below 0.001 (*p* = 0.0003). The ratio of the amplitudes in the two conditions, Att. F. vs. Att. M., was 0.8, or –2.2 dB.

The amplitude when attending the female speaker was also significantly lower than when reading the text, that is, when attention was focused on the visual modality, with a *p*-value below 0.001 (*p* = 0.0003, Figure 4J). In this case, the amplitude ratio was 0.7, or –2.6 dB. In contrast, no significant difference emerged when comparing the attended male voice (Att. M) condition to the attend visual condition (Att. V).

For the speech-like DPOAEs elicited by unresolved harmonics of the female voice, the stimulus *F*_unres_, we did not observe any difference in amplitudes across the three attentional conditions (Figure 4K).

For the male speaker, because the stimulus *M*_res_ did not give reliable speech-like DPOAEs, we could only assess the stimulus *M*_unres_ which utilized unresolved harmonics of the male voice. Significant amplitude differences emerged between all three conditions (Figure 4L). The amplitude when attending the male voice was significantly higher than in the other two conditions. The ratio between the amplitudes when attending the male voice and when attending the female one was 1.5, or 3.8 dB (*p* = 7x10^−7^). In addition, the amplitude when ignoring the male voice, i.e. attending the female voice, was smaller than when reading the text (ratio of 0.8, or –2 dB; *p* = 0.002). The amplitude difference between attending the male voice and reading the text was slightly less pronounced (*p* = 0.03) with a ratio of 1.2, or 1.7 dB.

For the delays of the speech-like DPOAEs, we did not find any significant differences between the three attentional conditions, in neither of the three stimuli (Figures 4M–O).

## 4 Discussion

This study examined the feasibility of simultaneously eliciting and measuring multiple speech-like DPOAEs in response to four pairs of stimuli composed of resolved and unresolved harmonics that were derived from two distinct voices. We further investigated whether the speech-like DPOAEs were modulated by selective auditory attention as well as by intermodal attention, and whether such attentional modulation differed between resolved and unresolved harmonics.

Our study shows that four speech-like DPOAEs in response to multiple stimuli pairs can be measured successfully. However, we observed considerable variability in the quality of the recorded responses between the different speech-like DPOAEs (cf. Figure 2), which may reflect either inherent variability in the generation mechanisms of these otoacoustic emissions or differences in noise levels due to varying frequencies of the eliciting stimulus waveforms. In addition, not all significant speech-like DPOAE peaks shared the same morphology; variations were observed in peak width, SNR, peak height, and peak latency.

While a previous study from our group already demonstrated the feasibility of recording speech-like DPOAEs (Saiz-Alía et al., 2021), here, we extend this approach by eliciting speech-like DPOAEs with four simultaneously presented harmonic pairs instead of only one, thereby increasing both the speech-related information in the stimulus and the resulting DPOAE signal, and more closely approximating natural speech.

Further, we selected the employed harmonics such that they were either clearly resolved or clearly unresolved, strengthening the interpretability of our conditions. Indeed, our previous work reported indications of attentional effects in speech-like DPOAEs, but also noted inconsistencies between the male and female voices (Saiz-Alía et al., 2021). These may be attributed to differences in the harmonic structures employed, with the harmonics of the male voice in the previous study occupying the transition zone between resolved and unresolved harmonics.

Importantly, the auditory and visual stimuli remained statistically the same throughout the different attentional conditions. In particular, both audio streams and the visual text were presented concurrently with all four stimulus pairs for eliciting the speech-like DPOAEs. As a result, the only variable that differed between conditions was the participant's attentional focus. This design minimized the likelihood of systematic confounds such as differences in movement or task structure. Furthermore, even if the middle-ear muscle reflex should have been activated despite the moderate stimulus volumes, it would have affected all attentional conditions equally.

Regarding the behavioral results, we found that the comprehension scores did not differ significantly across the attentional conditions, confirming that task difficulty was well-matched. Small differences in self-reported mental effort were likely attributable to speaker-specific factors such as intonation.

### 4.1 Frequency-dependency of speech-like DPOAEs

Our evaluation of the amplitude of the speech-like DPOAEs in the single-speaker scenario revealed a pronounced dependency on the emission frequency (Figure 3E). Consistent with previous findings on pure-tone DPOAEs, amplitudes were strongest for stimulus frequencies around 1 kHz (Probst et al., 1991). Both lower and higher emission frequencies resulted in lower amplitudes. Because noise in electronics as well as in mechanical and acoustic systems increases at lower frequencies, the signal at the lowest emission, around 350 Hz for the stimulus *M*_res_, could not be detected in most trials. This stimulus was therefore excluded from further analysis.

### 4.2 Attentional effects

We observed attentional modulation of the amplitudes of the speech-like DPOAEs for the stimuli *F*_res_ and *M*_unres_, but not for *F*_unres_.

The hypothesized differences between resolved and unresolved harmonics emerged clearly for the speech-like DPOAEs related to the female voice. Resolved harmonics, such as those employed for the stimulus *F*_res_, produce spatially distinct excitation peaks along the basilar membrane (Pit, 2005; Bernstein and Oxenham, 2003). Vibrations at these locations can be selectively enhanced through higher gain of the active process, which can help to enhance the neural representation of a target speech. In contrast, unresolved harmonics generate overlapping peaks along the basilar membrane, making such a spatial filter unfeasible. These considerations likely explain the presence of attentional modulation for the stimulus *F*_res_ together with its absence for the stimulus *F*_unres_.

The direction of the observed attentional effect for speech-like DPOAEs elicited by the stimulus *F*_res_, however, was unexpected: the resolved harmonics of the female voice counterintuitively caused *lower* amplitudes when attention was directed at the female voice versus when the male voice or the visual task was attended. Taken at face value, this result means that speech-like DPOAEs are weaker when a signal is attended than when it is ignored. This could suggest that the cochlea amplifies the harmonic structure of the target voice *less* than the background noise. Although unexpected, similar decreases have been reported in previous DPOAE studies (Smith et al., 2012; Srinivasan et al., 2012, 2014) and occasionally elsewhere in the auditory system [e.g., decreases in neural tracking when intelligibility is high (Hauswald et al., 2022)].

One plausible explanation for the observed reduction in speech-like DPOAE amplitudes during selective attention is rooted in the known operating principles of the medial olivocochlear (MOC) efferent system. If we suppose that attending to a target signal enhances MOC activity, it consequently suppresses outer hair cell–mediated cochlear amplification and thereby partially linearizes the basilar membrane input–output function by reducing its compressive nonlinearity (Maison et al., 2001; Guinan, 2006). Such partial linearization has been proposed to improve speech intelligibility in noisy environments by suppressing background energy more uniformly, while preserving the salience of structured speech components (Maison et al., 2001; Micheyl and Collet, 1993). Importantly, however, distortion product otoacoustic emissions rely on strong local nonlinearities of the BM response. Consequently, MOC-induced linearization may lead to reduced DPOAE generation, even in conditions where perceptual performance is enhanced.

Still, it should be noted that the present paradigm does not involve classical speech-in-noise, but rather a speech-in-speech scenario in which both the target and the competing signal exhibit similar spectral structure and density. It therefore remains unclear to what extent mechanisms proposed for broadband noise suppression generalize to this more specialized listening situation.

In this context, it is instructive to consider previous studies that reported heterogeneous outcomes. (Wittekindt et al. 2014) found reduced DPOAE levels during visual attention when comparing levels to a baseline value of inattention, but no change during auditory attention, whereas (Walsh et al. 2015) observed attentional differences whose directions varied from subject to subject. These findings were challenged by (Francis et al. 2018), who observed that switching between states of attention and inattention produced changes in ear-canal noise that could mimic modulation of otoacoustic emissions. Our design avoids this confound: both auditory streams and the visual text were presented in all three attentional conditions, and only the focus of attention varied. We did not measure states of inattention. Thus, ear-canal noise and participant movement were expected to be equal across trials.

Other findings add to the mixed picture. (Beim et al. 2018) and (Beim et al. 2019) reported higher SFOAEs during auditory attention in one study but failed to replicate the effect in a second cohort. Earlier work by (Michie et al. 1996) also found no attentional effect on tone-pip evoked OAEs. On the other hand, evidence for efferent modulation of otoacoustic emissions comes from a recent work showing that the predictability of tone sequences modulates DPOAE amplitudes depending on behavioral relevance (Riecke et al., 2020). Differences across study designs, types of OAEs, and susceptibility to noise likely contribute to these discrepancies.

The study most comparable to this is our earlier one on speech-like DPOAEs (Saiz-Alía et al., 2021). It found a positive attentional modulation coefficient for the female voice, indicating larger DPOAEs when the corresponding voice was attended. However, the reported effect was modest (*p* = 0.02) compared with the clearer differences observed here (*p* = 0.0003), and the actual DPOAE amplitudes did not differ significantly. Key methodological differences to this study include the previous use of only one harmonic pair per voice, partial placement of male harmonics in the resolved–unresolved transition region, and separate measurement blocks for attended and ignored states.

A possible origin of the weaker speech-like DPOAE when attending the corresponding voice may lie in the peculiar mechanics of the cochlea at low frequencies. Classical descriptions based on critical-layer absorption accurately capture basal, high-frequency processing (Lighthill, 1981; Robles and Ruggero, 2001; Reichenbach and Hudspeth, 2014), but several studies indicate that this framework may not apply straightforwardly at frequencies lower than 4 kHz (Shera et al., 2010; Siegel et al., 2005; Temchin et al., 2008; Ashmore, 2008).

In this low-frequency regime, previous studies have proposed an alternative mode of operation, including independent resonance of the active process and unidirectional coupling between the basilar membrane and outer hair cells (Reichenbach and Hudspeth, 2010, 2011). Such mechanisms could suppress backward-propagating distortion products and may therefore provide an explanation for the direction of the attentional effects observed in our data.

Alternatively, the observed reduction of speech-like DPOAE amplitudes for the attended voice may result from phase-dependent interference between different DPOAE generation mechanisms, such as distortion and coherent reflection sources, whose relative phase relationships may be altered by attentional state or stimulus context. Changes in these relationships could lead to partial phase cancellation at the recording site, resulting in an apparent suppression of the measured emission without a corresponding reduction in local cochlear nonlinearity.

Another potential contributor is activation of the middle ear muscle reflex (MEMR). Sensitive wideband measures indicate that the MEMR can be elicited by acoustic stimuli at levels substantially lower than clinical thresholds, with detectable effects as low as 60 dB SPL in some individuals and measurement paradigms, and strength that varies with stimulus level and prior acoustic context (Baricevich et al., 2025). However, there is no clear evidence that MEMR activation occurs at the moderate stimulus levels used here (~57 dB SPL). Still, a contribution of weak or transient MEMR activity to the measured emission amplitudes cannot be fully excluded. However, as all attentional conditions in the present study shared the same acoustic stimuli, any MEMR activation driven by overall stimulus context or task engagement would be expected to occur in a statistically similar manner across conditions. Under this assumption, MEMR-related attenuation would primarily contribute to an overall modification of speech-like DPOAE amplitude rather than to the systematic, condition-specific differences observed here.

The speech-like DPOAEs evoked by the *F*_res_ stimuli did not differ between the Att. M condition (equivalent to ignoring the female voice) and the Att. V condition. This suggests that cochlea activity at the resolved harmonics of an ignored speaker—in this case, the female voice—remains the same in these two conditions. One possibility, in line with the above considerations regarding apical cochlear mechanics, is that the resolved harmonics of a target speaker are enhanced through the active process, but yield lower speech-like DPOAEs, e.g., due to the peculiarities of low-frequency cochlear mechanics. When this voice is not attended, the harmonics are less enhanced, independent of whether attention is directed toward another auditory stream or the visual signal.

**Figure 5:**
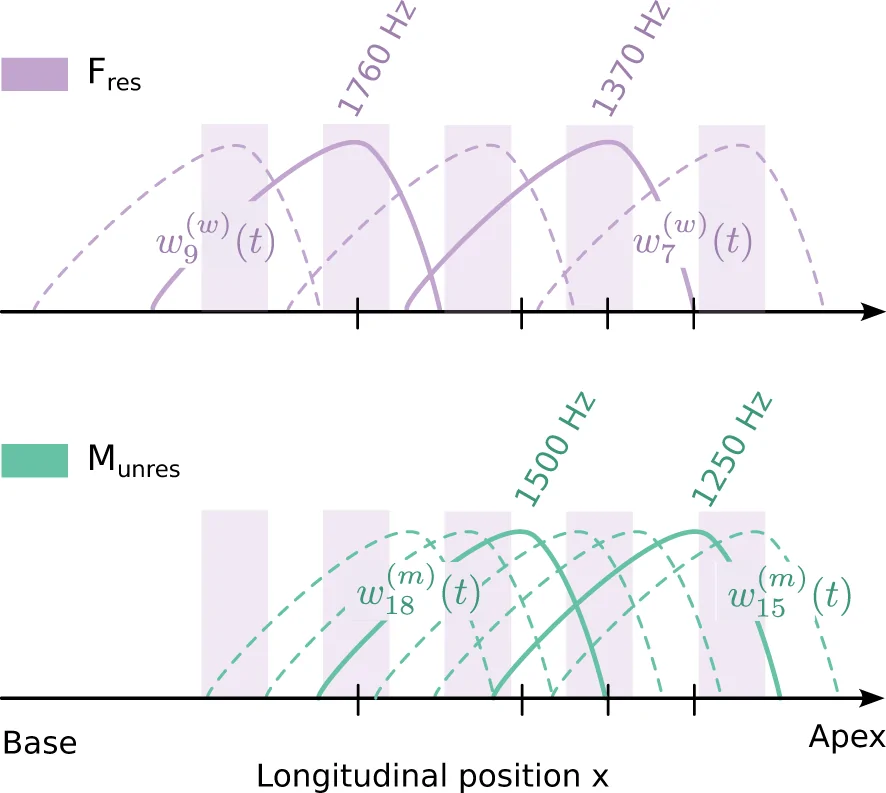
Resolved and unresolved harmonics. In the part of the cochlea where the *F*_res_ and the *M*_unres_ stimuli have their characteristic places, the harmonics of the female voice are resolved **(top)** while those of the male voice are unresolved **(bottom)**. The cochlear active process may enhance the response at the locations where the resolved harmonics of the female voice peak (purple shading). This enhancement will then also affect the responses to the unresolved harmonics of the male voice at these locations.

Because unresolved harmonics do not produce distinct peaks along the basilar membrane, the attentional modulation observed for the *M*_unres_ stimulus was unexpected. However, it is important to note that the *M*_unres_ stimulus was presented against a background of resolved harmonics of the female voice (Figure 5). We hypothesize that attention to the female voice enhances cochlear activity in the regions corresponding to its resolved harmonics (purple shading in Figure 5). This enhanced activity will also affect the responses to certain unresolved harmonics of the male voice, namely those that peak in the same section of the basilar membrane. These included the waveforms used in the *M*_unres_ stimulus. When the attentional focus changed from the female to the male voice, we hypothesize that cochlear activity in these regions became smaller, again affecting the corresponding unresolved harmonics of the male voice, including the *M*_unres_ stimulus. The attentional modulation for the *M*_unres_ stimulus was thus likely caused by changes in cochlear activity for the resolved harmonics of the female voice.

### 4.3 Delay of the speech-DPOAEs

The delays reported here do not represent classical DPOAE group delays derived from phase–frequency slopes. Instead, they reflect latency estimates obtained from the peak position of the cross-correlation between the expected speech-like DPOAE waveform and the recorded ear-canal signal. This time-domain measure can be interpreted as an approximation of the effective DPOAE latency, and thus allows qualitative comparison with known DPOAE delay behavior. As expected, these latency estimates did not depend on the focus of selective attention (Figures 4M–O). However, they differed systematically between stimulus types in the single-speaker condition due to frequency dependencies.

For the *F*_unres_ stimulus, speech-like DPOAEs peaked at approximately 0 ms, consistent with reports of near-zero DPOAE group delays at frequencies above ~1–1.5 kHz, where cochlear scaling symmetry and wave-fixed distortion generation are expected to hold (Faulstich and Kössl, 2000; Dhar et al., 2011). Similarly, the *M*_unres_ stimulus yielded short latencies of about 0.7 ms (approximately 0.7 cycles). Given that the average stimulus and DPOAE frequencies for this condition lie in the range of 1–1.5 kHz, this delay is consistent with a partial breakdown of scaling symmetry in this frequency region.

In contrast, speech-like DPOAEs evoked by the *F*_res_ stimulus peaked at approximately 2 ms, corresponding to about 2 stimulus cycles. Even though the frequency regime largely overlaps with that of the stimulus *M*_unres_, scaling symmetry seems to break down more fully, and the observed delay is compatible with distortion generation near the peak of the basilar-membrane traveling wave, followed by backward propagation through a slow basilar-membrane wave (Shera and Guinan, 1999; Tubis et al., 2000; Probst et al., 1991; Knight and Kemp, 2001; Robles and Ruggero, 2001).

The *M*_res_ stimulus did not produce reliable responses and was therefore excluded from further analysis.

### 4.4 Limitations

Comparability of the stimuli derived from the male voice was limited. In the competing speaker scenario, the stimulus *M*_res_ failed, in most trials, to produce significant speech-like DPOAEs for statistical evaluation. Additionally, the stimulus *M*_unres_ overlapped in frequency with the stimulus *F*_res_, compromising interpretability due to potential confounds in the origin of observed effects. Indeed, this overlap precludes a clear dissociation between modulation driven by harmonic resolvability and modulation driven by attention to a shared frequency region, thereby limiting the interpretability of the observed effects.

The conclusions drawn in the present study are specific to the frequency regions investigated by means of the F_*res*_ stimuli for resolved harmonics and of the F_*unres*_ stimuli for unresolved harmonics, spanning approximately 1–1.8 kHz and 2.3–3.5 kHz, respectively. While these two frequency ranges yield robust speech-like DPOAEs, it remains unclear to what degree the obtained results can be generalized to different frequency regions of the cochlea. In particular, the failure to obtain speech-like DPOAEs for the *M*_res_ stimulus at lower frequencies highlights the vulnerability of speech-like DPOAE measurements to low-frequency noise and reduced emission strength.

Future work is thus required to extend the present paradigm to additional frequency bands, with stimulus designs optimized for higher and lower cochlear regions and special focus on avoiding spectral overlap, in order to assess the frequency dependence and generalizability of attentional effects on speech-like DPOAEs.

### 4.5 Conclusion

Our study demonstrates that selective attention modulates the morphology of speech-like DPOAEs elicited by multiple, simultaneously presented harmonic pairs derived from natural speech signals. These findings are consistent with the notion that DPOAEs can also be evoked by natural, running speech, although extracting such responses remains challenging due to the high noise floor and spectral complexity of real speech. Within these constraints, the experimental paradigm employed here provides a tractable approximation for probing cochlear responses to ecologically relevant stimuli.

Attentional effects were observed for stimuli derived from both resolved and unresolved harmonic regions. Given the partial overlap in frequency content between conditions, we argue that the effects seen for the unresolved harmonics most likely resulted from attentional modulation of the resolved harmonics of the competing speaker. Notably, the direction and pattern of attentional modulation were consistent, whether attention was shifted from the target speech to a competing voice or from the target to a visual task. However, further work is required to unequivocally establish the attentional effects on resolved and unresolved harmonics.

Unexpectedly, attention reduced—rather than enhanced—the speech-like DPOAE associated with the attended speaker. In light of heterogeneous findings in the existing literature, replication and systematic extension of this effect across frequency regions and stimulus configurations will be necessary to establish its robustness and generality.

More broadly, we hope that the speech-like DPOAE paradigm introduced here will contribute to a deeper understanding of how the active cochlea participates in the perceptual and cognitive processing of complex naturalistic signals such as speech.

## Data Availability

All custom code used for stimulus generation, speech-like DPOAE analysis via cross-correlation, and statistical evaluation is openly available on GitHub at https://github.com/janna-stb/dpoae_attention_study.git under the MIT License.
